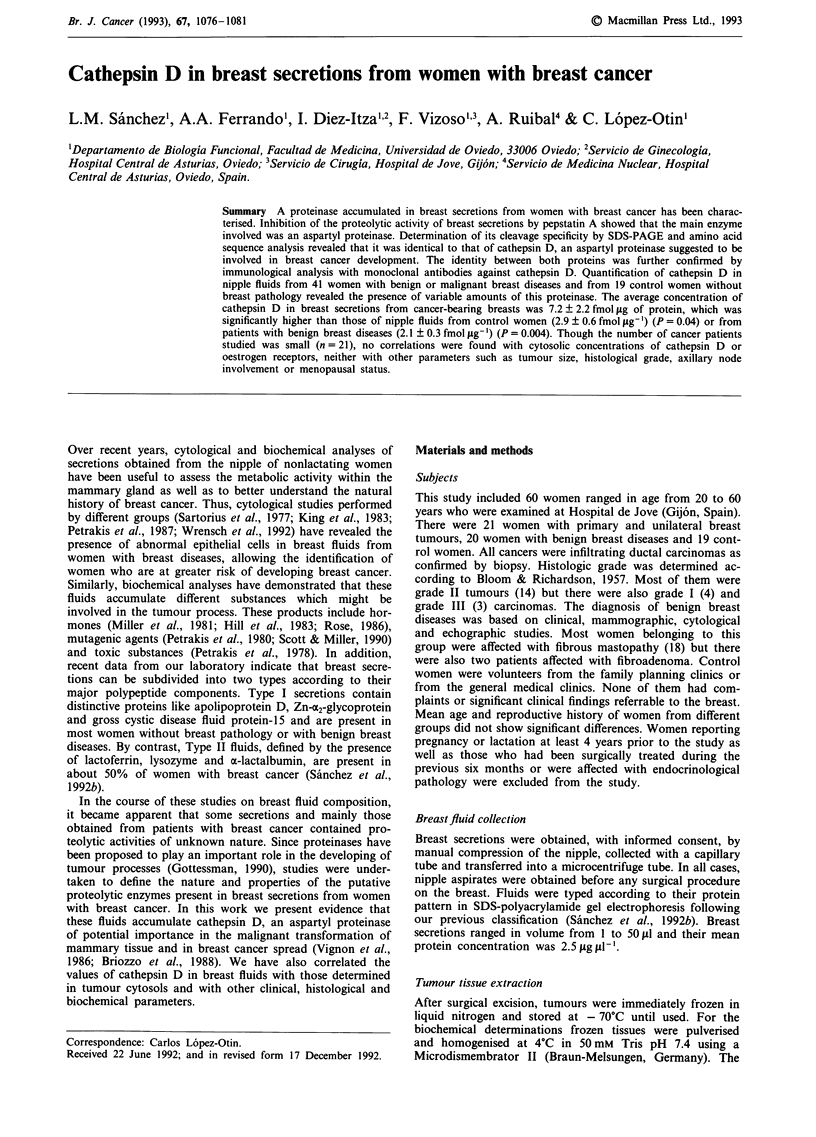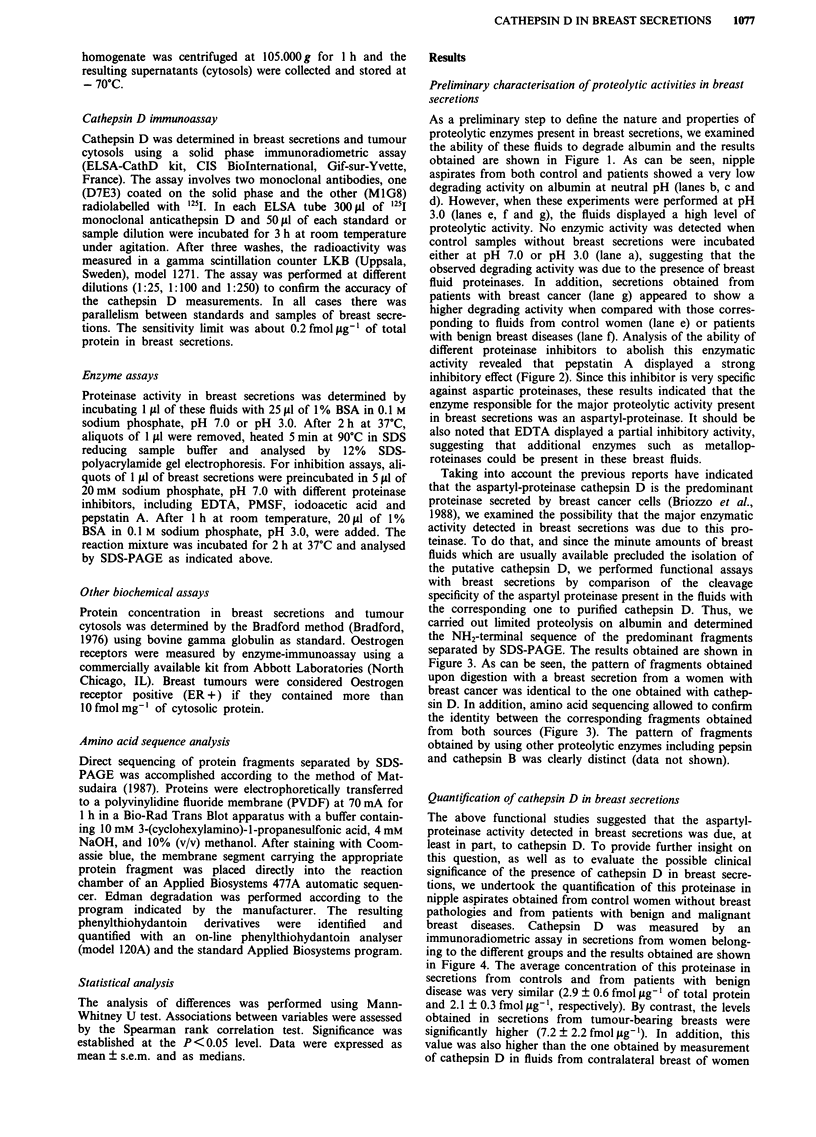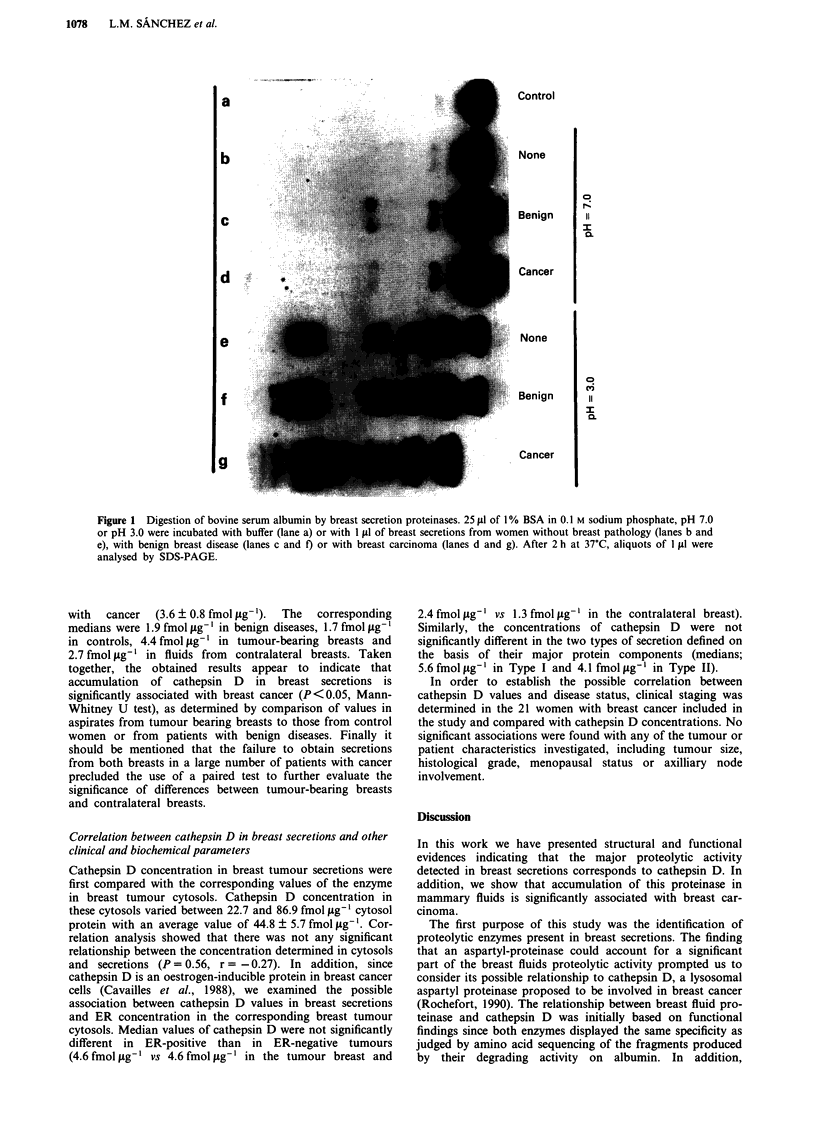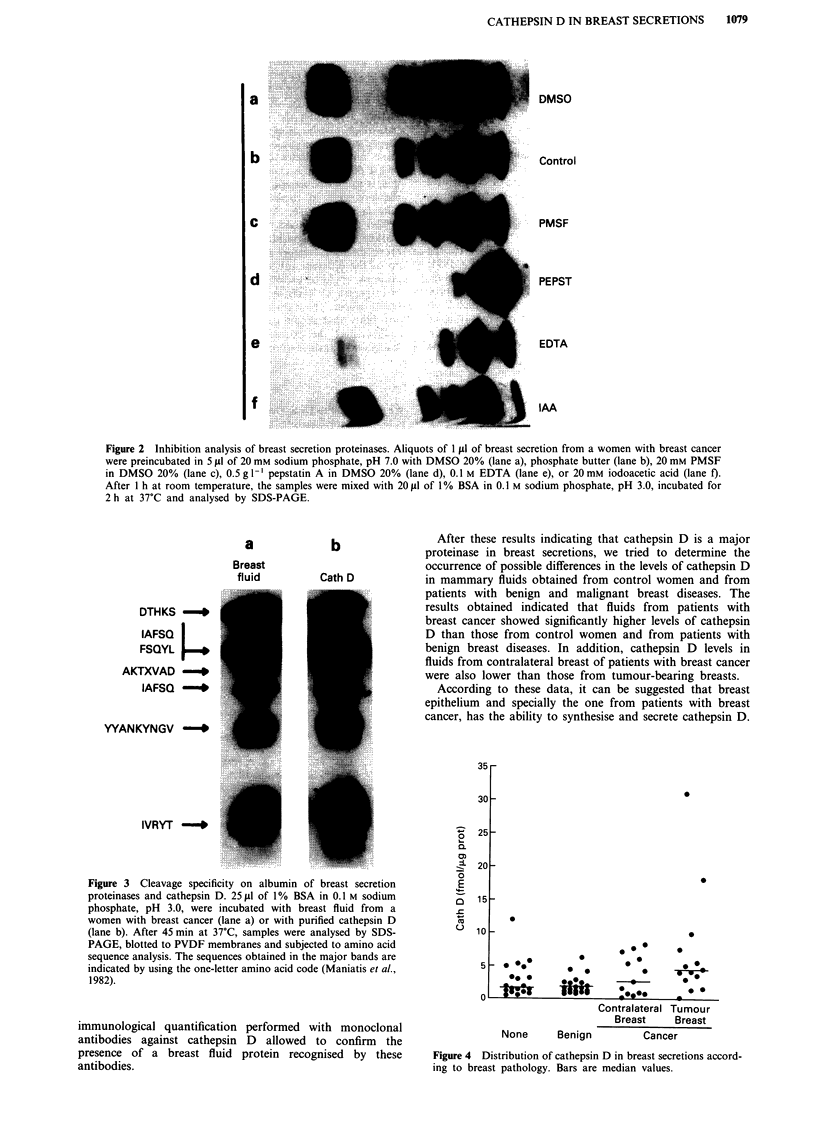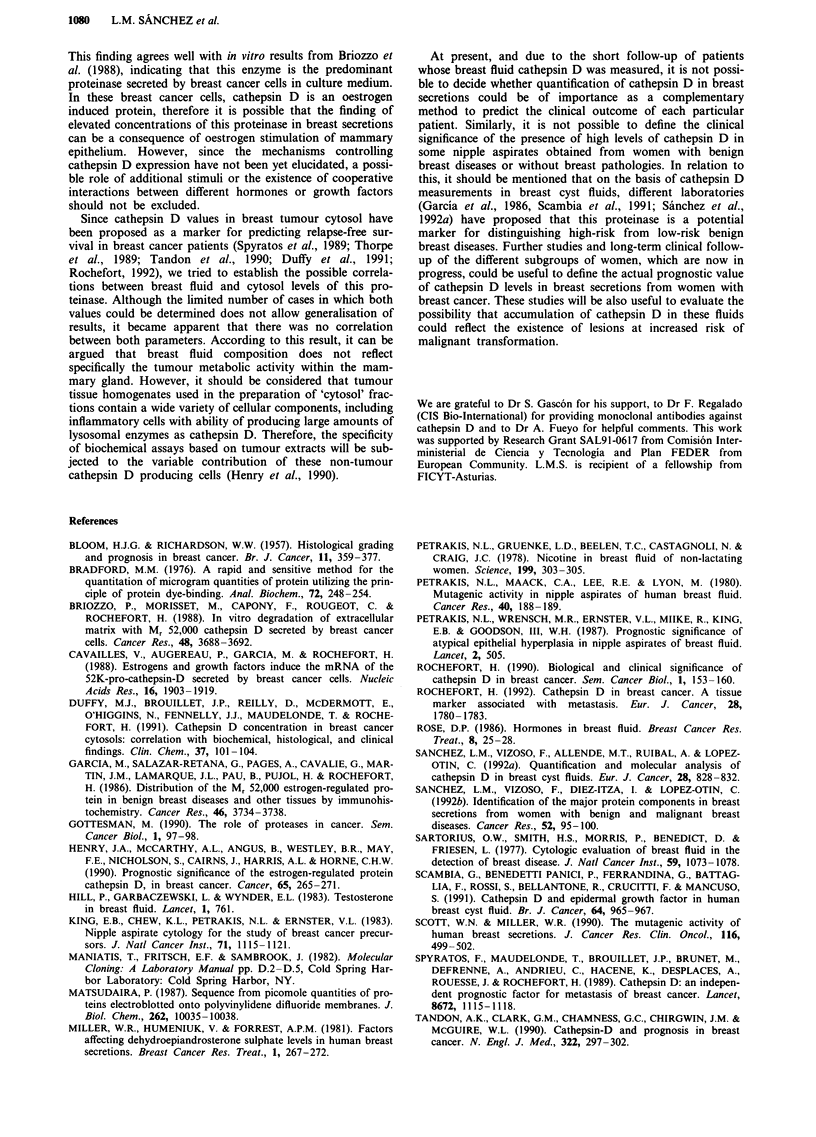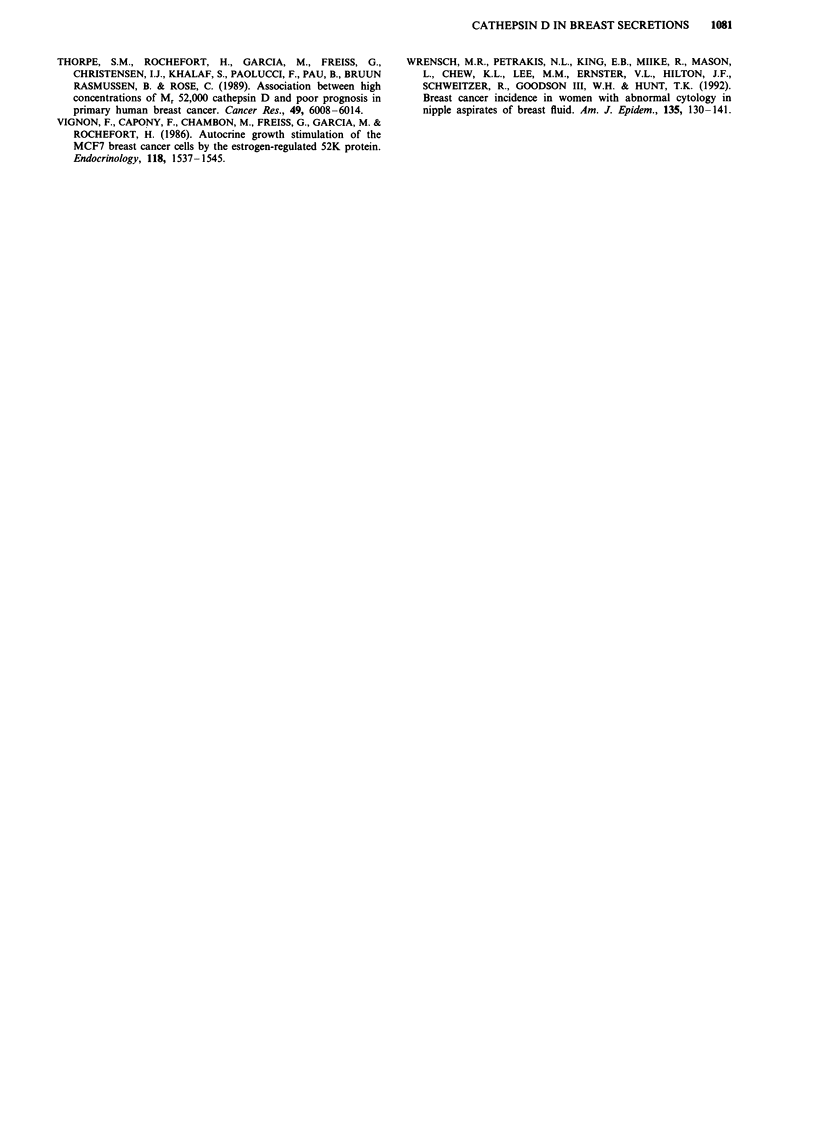# Cathepsin D in breast secretions from women with breast cancer.

**DOI:** 10.1038/bjc.1993.197

**Published:** 1993-05

**Authors:** L. M. Sánchez, A. A. Ferrando, I. Diez-Itza, F. Vizoso, A. Ruibal, C. López-Otin

**Affiliations:** Departamento de Biologia Funcional, Facultad de Medicina, Universidad de Oviedo, Spain.

## Abstract

**Images:**


*Br. J. Cancer* (1993), **67**, 1076–1081 © Macmillan Press Ltd., 1993

### Cathepsin D in breast secretions from women with breast cancer

L.M. Sánchez[1], A.A. Ferrando[1], I. Diez-Itza[1,2], F. Vizoso[1,3], A. Ruibal[4] & C. López-Otin[1]

[1]*Departamento de Biologia Funcional, Facultad de Medicina, Universidad de Oviedo, 33006 Oviedo;* [2]*Servicio de Ginecologia, Hospital Central de Asturias, Oviedo;* [3]*Servicio de Cirugia, Hospital de Jove, Gijón;* [4]*Servicio de Medicina Nuclear, Hospital Central de Asturias, Oviedo, Spain.*

**Summary** A proteinase accumulated in breast secretions from women with breast cancer has been characterised. Inhibition of the proteolytic activity of breast secretions by pepstatin A showed that the main enzyme involved was an aspartyl proteinase. Determination of its cleavage specificity by SDS-PAGE and amino acid sequence analysis revealed that it was identical to that of cathepsin D, an aspartyl proteinase suggested to be involved in breast cancer development. The identity between both proteins was further confirmed by immunological analysis with monoclonal antibodies against cathepsin D. Quantification of cathepsin D in nipple fluids from 41 women with benign or malignant breast diseases and from 19 control women without breast pathology revealed the presence of variable amounts of this proteinase. The average concentration of cathepsin D in breast secretions from cancer-bearing breasts was 7.2 ± 2.2 fmol μg of protein, which was significantly higher than those of nipple fluids from control women (2.9 ± 0.6 fmol $\mu g^{-1}$) ($P = 0.04$) or from patients with benign breast diseases (2.1 ± 0.3 fmol $\mu g^{-1}$) ($P = 0.004$). Though the number of cancer patients studied was small ($n = 21$), no correlations were found with cytosolic concentrations of cathepsin D or oestrogen receptors, neither with other parameters such as tumour size, histological grade, axillary node involvement or menopausal status.

Over recent years, cytological and biochemical analyses of secretions obtained from the nipple of nonlactating women have been useful to assess the metabolic activity within the mammary gland as well as to better understand the natural history of breast cancer. Thus, cytological studies performed by different groups (Sartorius *et al.*, 1977; King *et al.*, 1983; Petrakis *et al.*, 1987; Wrensch *et al.*, 1992) have revealed the presence of abnormal epithelial cells in breast fluids from women with breast diseases, allowing the identification of women who are at greater risk of developing breast cancer. Similarly, biochemical analyses have demonstrated that these fluids accumulate different substances which might be involved in the tumour process. These products include hormones (Miller *et al.*, 1981; Hill *et al.*, 1983; Rose, 1986), mutagenic agents (Petrakis *et al.*, 1980; Scott & Miller, 1990) and toxic substances (Petrakis *et al.*, 1978). In addition, recent data from our laboratory indicate that breast secretions can be subdivided into two types according to their major polypeptide components. Type I secretions contain distinctive proteins like apolipoprotein D, Zn-$\alpha_2$-glycoprotein and gross cystic disease fluid protein-15 and are present in most women without breast pathology or with benign breast diseases. By contrast, Type II fluids, defined by the presence of lactoferrin, lysozyme and α-lactalbumin, are present in about 50% of women with breast cancer (Sánchez *et al.*, 1992*b*).

In the course of these studies on breast fluid composition, it became apparent that some secretions and mainly those obtained from patients with breast cancer contained proteolytic activities of unknown nature. Since proteinases have been proposed to play an important role in the developing of tumour processes (Gottessman, 1990), studies were undertaken to define the nature and properties of the putative proteolytic enzymes present in breast secretions from women with breast cancer. In this work we present evidence that these fluids accumulate cathepsin D, an aspartyl proteinase of potential importance in the malignant transformation of mammary tissue and in breast cancer spread (Vignon *et al.*, 1986; Briozzo *et al.*, 1988). We have also correlated the values of cathepsin D in breast fluids with those determined in tumour cytosols and with other clinical, histological and biochemical parameters.

Correspondence: Carlos López-Otin.
Received 22 June 1992; and in revised form 17 December 1992.

### Materials and methods

### *Subjects*

This study included 60 women ranged in age from 20 to 60 years who were examined at Hospital de Jove (Gijón, Spain). There were 21 women with primary and unilateral breast tumours, 20 women with benign breast diseases and 19 control women. All cancers were infiltrating ductal carcinomas as confirmed by biopsy. Histologic grade was determined according to Bloom & Richardson, 1957. Most of them were grade II tumours (14) but there were also grade I (4) and grade III (3) carcinomas. The diagnosis of benign breast diseases was based on clinical, mammographic, cytological and echographic studies. Most women belonging to this group were affected with fibrous mastopathy (18) but there were also two patients affected with fibroadenoma. Control women were volunteers from the family planning clinics or from the general medical clinics. None of them had complaints or significant clinical findings referrable to the breast. Mean age and reproductive history of women from different groups did not show significant differences. Women reporting pregnancy or lactation at least 4 years prior to the study as well as those who had been surgically treated during the previous six months or were affected with endocrinological pathology were excluded from the study.

### *Breast fluid collection*

Breast secretions were obtained, with informed consent, by manual compression of the nipple, collected with a capillary tube and transferred into a microcentrifuge tube. In all cases, nipple aspirates were obtained before any surgical procedure on the breast. Fluids were typed according to their protein pattern in SDS-polyacrylamide gel electrophoresis following our previous classification (Sánchez *et al.*, 1992*b*). Breast secretions ranged in volume from 1 to 50 μl and their mean protein concentration was 2.5 μg $\mu l^{-1}$.

### *Tumour tissue extraction*

After surgical excision, tumours were immediately frozen in liquid nitrogen and stored at − 70°C until used. For the biochemical determinations frozen tissues were pulverised and homogenised at 4°C in 50 mM Tris pH 7.4 using a Microdismembrator II (Braun-Melsungen, Germany). The homogenate was centrifuged at 105.000 $g$ for 1 h and the resulting supernatants (cytosols) were collected and stored at − 70°C.

*Cathepsin D immunoassay*

Cathepsin D was determined in breast secretions and tumour cytosols using a solid phase immunoradiometric assay (ELSA-CathD kit, CIS BioInternational, Gif-sur-Yvette, France). The assay involves two monoclonal antibodies, one (D7E3) coated on the solid phase and the other (M1G8) radiolabelled with $^{125}I$. In each ELSA tube 300 µl of $^{125}I$ monoclonal anticathepsin D and 50 µl of each standard or sample dilution were incubated for 3 h at room temperature under agitation. After three washes, the radioactivity was measured in a gamma scintillation counter LKB (Uppsala, Sweden), model 1271. The assay was performed at different dilutions (1:25, 1:100 and 1:250) to confirm the accuracy of the cathepsin D measurements. In all cases there was parallelism between standards and samples of breast secretions. The sensitivity limit was about 0.2 fmol $\mu g^{-1}$ of total protein in breast secretions.

*Enzyme assays*

Proteinase activity in breast secretions was determined by incubating 1 µl of these fluids with 25 µl of 1% BSA in 0.1 M sodium phosphate, pH 7.0 or pH 3.0. After 2 h at 37°C, aliquots of 1 µl were removed, heated 5 min at 90°C in SDS reducing sample buffer and analysed by 12% SDS-polyacrylamide gel electrophoresis. For inhibition assays, aliquots of 1 µl of breast secretions were preincubated in 5 µl of 20 mM sodium phosphate, pH 7.0 with different proteinase inhibitors, including EDTA, PMSF, iodoacetic acid and pepstatin A. After 1 h at room temperature, 20 µl of 1% BSA in 0.1 M sodium phosphate, pH 3.0, were added. The reaction mixture was incubated for 2 h at 37°C and analysed by SDS-PAGE as indicated above.

*Other biochemical assays*

Protein concentration in breast secretions and tumour cytosols was determined by the Bradford method (Bradford, 1976) using bovine gamma globulin as standard. Oestrogen receptors were measured by enzyme-immunoassay using a commercially available kit from Abbott Laboratories (North Chicago, IL). Breast tumours were considered Oestrogen receptor positive (ER+) if they contained more than 10 fmol $mg^{-1}$ of cytosolic protein.

*Amino acid sequence analysis*

Direct sequencing of protein fragments separated by SDS-PAGE was accomplished according to the method of Matsudaira (1987). Proteins were electrophoretically transferred to a polyvinylidine fluoride membrane (PVDF) at 70 mA for 1 h in a Bio-Rad Trans Blot apparatus with a buffer containing 10 mM 3-(cyclohexylamino)-1-propanesulfonic acid, 4 mM NaOH, and 10% (v/v) methanol. After staining with Coomassie blue, the membrane segment carrying the appropriate protein fragment was placed directly into the reaction chamber of an Applied Biosystems 477A automatic sequencer. Edman degradation was performed according to the program indicated by the manufacturer. The resulting phenylthiohydantoin derivatives were identified and quantified with an on-line phenylthiohydantoin analyser (model 120A) and the standard Applied Biosystems program.

*Statistical analysis*

The analysis of differences was performed using Mann-Whitney U test. Associations between variables were assessed by the Spearman rank correlation test. Significance was established at the $P<0.05$ level. Data were expressed as mean ± s.e.m. and as medians.

### Results

*Preliminary characterisation of proteolytic activities in breast secretions*

As a preliminary step to define the nature and properties of proteolytic enzymes present in breast secretions, we examined the ability of these fluids to degrade albumin and the results obtained are shown in Figure 1. As can be seen, nipple aspirates from both control and patients showed a very low degrading activity on albumin at neutral pH (lanes b, c and d). However, when these experiments were performed at pH 3.0 (lanes e, f and g), the fluids displayed a high level of proteolytic activity. No enzymic activity was detected when control samples without breast secretions were incubated either at pH 7.0 or pH 3.0 (lane a), suggesting that the observed degrading activity was due to the presence of breast fluid proteinases. In addition, secretions obtained from patients with breast cancer (lane g) appeared to show a higher degrading activity when compared with those corresponding to fluids from control women (lane e) or patients with benign breast diseases (lane f). Analysis of the ability of different proteinase inhibitors to abolish this enzymatic activity revealed that pepstatin A displayed a strong inhibitory effect (Figure 2). Since this inhibitor is very specific against aspartic proteinases, these results indicated that the enzyme responsible for the major proteolytic activity present in breast secretions was an aspartyl-proteinase. It should be also noted that EDTA displayed a partial inhibitory activity, suggesting that additional enzymes such as metalloproteinases could be present in these breast fluids.

Taking into account the previous reports have indicated that the aspartyl-proteinase cathepsin D is the predominant proteinase secreted by breast cancer cells (Briozzo *et al.*, 1988), we examined the possibility that the major enzymatic activity detected in breast secretions was due to this proteinase. To do that, and since the minute amounts of breast fluids which are usually available precluded the isolation of the putative cathepsin D, we performed functional assays with breast secretions by comparison of the cleavage specificity of the aspartyl proteinase present in the fluids with the corresponding one to purified cathepsin D. Thus, we carried out limited proteolysis on albumin and determined the $NH_2$-terminal sequence of the predominant fragments separated by SDS-PAGE. The results obtained are shown in Figure 3. As can be seen, the pattern of fragments obtained upon digestion with a breast secretion from a women with breast cancer was identical to the one obtained with cathepsin D. In addition, amino acid sequencing allowed to confirm the identity between the corresponding fragments obtained from both sources (Figure 3). The pattern of fragments obtained by using other proteolytic enzymes including pepsin and cathepsin B was clearly distinct (data not shown).

*Quantification of cathepsin D in breast secretions*

The above functional studies suggested that the aspartyl-proteinase activity detected in breast secretions was due, at least in part, to cathepsin D. To provide further insight on this question, as well as to evaluate the possible clinical significance of the presence of cathepsin D in breast secretions, we undertook the quantification of this proteinase in nipple aspirates obtained from control women without breast pathologies and from patients with benign and malignant breast diseases. Cathepsin D was measured by an immunoradiometric assay in secretions from women belonging to the different groups and the results obtained are shown in Figure 4. The average concentration of this proteinase in secretions from controls and from patients with benign disease was very similar (2.9 ± 0.6 fmol $\mu g^{-1}$ of total protein and 2.1 ± 0.3 fmol $\mu g^{-1}$, respectively). By contrast, the levels obtained in secretions from tumour-bearing breasts were significantly higher (7.2 ± 2.2 fmol $\mu g^{-1}$). In addition, this value was also higher than the one obtained by measurement of cathepsin D in fluids from contralateral breast of women

**Figure 1** Digestion of bovine serum albumin by breast secretion proteinases. 25 µl of 1% BSA in 0.1 M sodium phosphate, pH 7.0 or pH 3.0 were incubated with buffer (lane a) or with 1 µl of breast secretions from women without breast pathology (lanes b and e), with benign breast disease (lanes c and f) or with breast carcinoma (lanes d and g). After 2 h at 37°C, aliquots of 1 µl were analysed by SDS-PAGE.

with cancer ($3.6 \pm 0.8$ fmol $\mu g^{-1}$). The corresponding medians were 1.9 fmol $\mu g^{-1}$ in benign diseases, 1.7 fmol $\mu g^{-1}$ in controls, 4.4 fmol $\mu g^{-1}$ in tumour-bearing breasts and 2.7 fmol $\mu g^{-1}$ in fluids from contralateral breasts. Taken together, the obtained results appear to indicate that accumulation of cathepsin D in breast secretions is significantly associated with breast cancer ($P<0.05$, Mann-Whitney U test), as determined by comparison of values in aspirates from tumour bearing breasts to those from control women or from patients with benign diseases. Finally it should be mentioned that the failure to obtain secretions from both breasts in a large number of patients with cancer precluded the use of a paired test to further evaluate the significance of differences between tumour-bearing breasts and contralateral breasts.

### *Correlation between cathepsin D in breast secretions and other clinical and biochemical parameters*

Cathepsin D concentration in breast tumour secretions were first compared with the corresponding values of the enzyme in breast tumour cytosols. Cathepsin D concentration in these cytosols varied between 22.7 and 86.9 fmol $\mu g^{-1}$ cytosol protein with an average value of $44.8 \pm 5.7$ fmol $\mu g^{-1}$. Correlation analysis showed that there was not any significant relationship between the concentration determined in cytosols and secretions ($P = 0.56$, $r = -0.27$). In addition, since cathepsin D is an oestrogen-inducible protein in breast cancer cells (Cavailles *et al.*, 1988), we examined the possible association between cathepsin D values in breast secretions and ER concentration in the corresponding breast tumour cytosols. Median values of cathepsin D were not significantly different in ER-positive than in ER-negative tumours (4.6 fmol $\mu g^{-1}$ *vs* 4.6 fmol $\mu g^{-1}$ in the tumour breast and 2.4 fmol $\mu g^{-1}$ *vs* 1.3 fmol $\mu g^{-1}$ in the contralateral breast). Similarly, the concentrations of cathepsin D were not significantly different in the two types of secretion defined on the basis of their major protein components (medians; 5.6 fmol $\mu g^{-1}$ in Type I and 4.1 fmol $\mu g^{-1}$ in Type II).

In order to establish the possible correlation between cathepsin D values and disease status, clinical staging was determined in the 21 women with breast cancer included in the study and compared with cathepsin D concentrations. No significant associations were found with any of the tumour or patient characteristics investigated, including tumour size, histological grade, menopausal status or axilliary node involvement.

### Discussion

In this work we have presented structural and functional evidences indicating that the major proteolytic activity detected in breast secretions corresponds to cathepsin D. In addition, we show that accumulation of this proteinase in mammary fluids is significantly associated with breast carcinoma.

The first purpose of this study was the identification of proteolytic enzymes present in breast secretions. The finding that an aspartyl-proteinase could account for a significant part of the breast fluids proteolytic activity prompted us to consider its possible relationship to cathepsin D, a lysosomal aspartyl proteinase proposed to be involved in breast cancer (Rochefort, 1990). The relationship between breast fluid proteinase and cathepsin D was initially based on functional findings since both enzymes displayed the same specificity as judged by amino acid sequencing of the fragments produced by their degrading activity on albumin. In addition,

**Figure 2** Inhibition analysis of breast secretion proteinases. Aliquots of 1 μl of breast secretion from a women with breast cancer were preincubated in 5 μl of 20 mM sodium phosphate, pH 7.0 with DMSO 20% (lane a), phosphate butter (lane b), 20 mM PMSF in DMSO 20% (lane c), 0.5 g $l^{-1}$ pepstatin A in DMSO 20% (lane d), 0.1 M EDTA (lane e), or 20 mM iodoacetic acid (lane f). After 1 h at room temperature, the samples were mixed with 20 μl of 1% BSA in 0.1 M sodium phosphate, pH 3.0, incubated for 2 h at 37°C and analysed by SDS-PAGE.

**Figure 3** Cleavage specificity on albumin of breast secretion proteinases and cathepsin D. 25 μl of 1% BSA in 0.1 M sodium phosphate, pH 3.0, were incubated with breast fluid from a women with breast cancer (lane a) or with purified cathepsin D (lane b). After 45 min at 37°C, samples were analysed by SDS-PAGE, blotted to PVDF membranes and subjected to amino acid sequence analysis. The sequences obtained in the major bands are indicated by using the one-letter amino acid code (Maniatis *et al*., 1982).

immunological quantification performed with monoclonal antibodies against cathepsin D allowed to confirm the presence of a breast fluid protein recognised by these antibodies.

After these results indicating that cathepsin D is a major proteinase in breast secretions, we tried to determine the occurrence of possible differences in the levels of cathepsin D in mammary fluids obtained from control women and from patients with benign and malignant breast diseases. The results obtained indicated that fluids from patients with breast cancer showed significantly higher levels of cathepsin D than those from control women and from patients with benign breast diseases. In addition, cathepsin D levels in fluids from contralateral breast of patients with breast cancer were also lower than those from tumour-bearing breasts.

According to these data, it can be suggested that breast epithelium and specially the one from patients with breast cancer, has the ability to synthesise and secrete cathepsin D.

**Figure 4** Distribution of cathepsin D in breast secretions according to breast pathology. Bars are median values.

This finding agrees well with *in vitro* results from Briozzo et al. (1988), indicating that this enzyme is the predominant proteinase secreted by breast cancer cells in culture medium. In these breast cancer cells, cathepsin D is an oestrogen induced protein, therefore it is possible that the finding of elevated concentrations of this proteinase in breast secretions can be a consequence of oestrogen stimulation of mammary epithelium. However, since the mechanisms controlling cathepsin D expression have not been yet elucidated, a possible role of additional stimuli or the existence of cooperative interactions between different hormones or growth factors should not be excluded.

Since cathepsin D values in breast tumour cytosol have been proposed as a marker for predicting relapse-free survival in breast cancer patients (Spyratos *et al.*, 1989; Thorpe *et al.*, 1989; Tandon *et al.*, 1990; Duffy *et al.*, 1991; Rochefort, 1992), we tried to establish the possible correlations between breast fluid and cytosol levels of this proteinase. Although the limited number of cases in which both values could be determined does not allow generalisation of results, it became apparent that there was no correlation between both parameters. According to this result, it can be argued that breast fluid composition does not reflect specifically the tumour metabolic activity within the mammary gland. However, it should be considered that tumour tissue homogenates used in the preparation of 'cytosol' fractions contain a wide variety of cellular components, including inflammatory cells with ability of producing large amounts of lysosomal enzymes as cathepsin D. Therefore, the specificity of biochemical assays based on tumour extracts will be subjected to the variable contribution of these non-tumour cathepsin D producing cells (Henry *et al.*, 1990).

At present, and due to the short follow-up of patients whose breast fluid cathepsin D was measured, it is not possible to decide whether quantification of cathepsin D in breast secretions could be of importance as a complementary method to predict the clinical outcome of each particular patient. Similarly, it is not possible to define the clinical significance of the presence of high levels of cathepsin D in some nipple aspirates obtained from women with benign breast diseases or without breast pathologies. In relation to this, it should be mentioned that on the basis of cathepsin D measurements in breast cyst fluids, different laboratories (García *et al.*, 1986, Scambia *et al.*, 1991; Sánchez *et al.*, 1992*a*) have proposed that this proteinase is a potential marker for distinguishing high-risk from low-risk benign breast diseases. Further studies and long-term clinical follow-up of the different subgroups of women, which are now in progress, could be useful to define the actual prognostic value of cathepsin D levels in breast secretions from women with breast cancer. These studies will be also useful to evaluate the possibility that accumulation of cathepsin D in these fluids could reflect the existence of lesions at increased risk of malignant transformation.

We are grateful to Dr S. Gascón for his support, to Dr F. Regalado (CIS Bio-International) for providing monoclonal antibodies against cathepsin D and to Dr A. Fueyo for helpful comments. This work was supported by Research Grant SAL91-0617 from Comisión Interministerial de Ciencia y Tecnología and Plan FEDER from European Community. L.M.S. is recipient of a fellowship from FICYT-Asturias.

### References

BLOOM, H.J.G. & RICHARDSON, W.W. (1957). Histological grading and prognosis in breast cancer. *Br. J. Cancer*, **11**, 359–377.

BRADFORD, M.M. (1976). A rapid and sensitive method for the quantitation of microgram quantities of protein utilizing the principle of protein dye-binding. *Anal. Biochem.*, **72**, 248–254.

BRIOZZO, P., MORISSET, M., CAPONY, F., ROUGEOT, C. & ROCHEFORT, H. (1988). In vitro degradation of extracellular matrix with $M_r$ 52,000 cathepsin D secreted by breast cancer cells. *Cancer Res.*, **48**, 3688–3692.

CAVAILLES, V., AUGEREAU, P., GARCIA, M. & ROCHEFORT, H. (1988). Estrogens and growth factors induce the mRNA of the 52K-pro-cathepsin-D secreted by breast cancer cells. *Nucleic Acids Res.*, **16**, 1903–1919.

DUFFY, M.J., BROUILLET, J.P., REILLY, D., MCDERMOTT, E., O'HIGGINS, N., FENNELLY, J.J., MAUDELONDE, T. & ROCHEFORT, H. (1991). Cathepsin D concentration in breast cancer cytosols: correlation with biochemical, histological, and clinical findings. *Clin. Chem.*, **37**, 101–104.

GARCIA, M., SALAZAR-RETANA, G., PAGES, A., CAVALIE, G., MARTIN, J.M., LAMARQUE, J.L., PAU, B., PUJOL, H. & ROCHEFORT, H. (1986). Distribution of the $M_r$ 52,000 estrogen-regulated protein in benign breast diseases and other tissues by immunohistochemistry. *Cancer Res.*, **46**, 3734–3738.

GOTTESMAN, M. (1990). The role of proteases in cancer. *Sem. Cancer Biol.*, **1**, 97–98.

HENRY, J.A., MCCARTHY, A.L., ANGUS, B., WESTLEY, B.R., MAY, F.E., NICHOLSON, S., CAIRNS, J., HARRIS, A.L. & HORNE, C.H.W. (1990). Prognostic significance of the estrogen-regulated protein cathepsin D, in breast cancer. *Cancer*, **65**, 265–271.

HILL, P., GARBACZEWSKI, L. & WYNDER, E.L. (1983). Testosterone in breast fluid. *Lancet*, **1**, 761.

KING, E.B., CHEW, K.L., PETRAKIS, N.L. & ERNSTER, V.L. (1983). Nipple aspirate cytology for the study of breast cancer precursors. *J. Natl Cancer Inst.*, **71**, 1115–1121.

MANIATIS, T., FRITSCH, E.F. & SAMBROOK, J. (1982). *Molecular Cloning: A Laboratory Manual* pp. D.2–D.5, Cold Spring Harbor Laboratory: Cold Spring Harbor, NY.

MATSUDAIRA, P. (1987). Sequence from picomole quantities of proteins electroblotted onto polyvinylidene difluoride membranes. *J. Biol. Chem.*, **262**, 10035–10038.

MILLER, W.R., HUMENIUK, V. & FORREST, A.P.M. (1981). Factors affecting dehydroepiandrosterone sulphate levels in human breast secretions. *Breast Cancer Res. Treat.*, **1**, 267–272.

PETRAKIS, N.L., GRUENKE, L.D., BEELEN, T.C., CASTAGNOLI, N. & CRAIG, J.C. (1978). Nicotine in breast fluid of non-lactating women. *Science*, **199**, 303–305.

PETRAKIS, N.L., MAACK, C.A., LEE, R.E. & LYON, M. (1980). Mutagenic activity in nipple aspirates of human breast fluid. *Cancer Res.*, **40**, 188–189.

PETRAKIS, N.L., WRENSCH, M.R., ERNSTER, V.L., MIIKE, R., KING, E.B. & GOODSON, III, W.H. (1987). Prognostic significance of atypical epithelial hyperplasia in nipple aspirates of breast fluid. *Lancet*, **2**, 505.

ROCHEFORT, H. (1990). Biological and clinical significance of cathepsin D in breast cancer. *Sem. Cancer Biol.*, **1**, 153–160.

ROCHEFORT, H. (1992). Cathepsin D in breast cancer. A tissue marker associated with metastasis. *Eur. J. Cancer*, **28**, 1780–1783.

ROSE, D.P. (1986). Hormones in breast fluid. *Breast Cancer Res. Treat.*, **8**, 25–28.

SANCHEZ, L.M., VIZOSO, F., ALLENDE, M.T., RUIBAL, A. & LOPEZ-OTIN, C. (1992*a*). Quantification and molecular analysis of cathepsin D in breast cyst fluids. *Eur. J. Cancer*, **28**, 828–832.

SANCHEZ, L.M., VIZOSO, F., DIEZ-ITZA, I. & LOPEZ-OTIN, C. (1992*b*). Identification of the major protein components in breast secretions from women with benign and malignant breast diseases. *Cancer Res.*, **52**, 95–100.

SARTORIUS, O.W., SMITH, H.S., MORRIS, P., BENEDICT, D. & FRIESEN, L. (1977). Cytologic evaluation of breast fluid in the detection of breast disease. *J. Natl Cancer Inst.*, **59**, 1073–1078.

SCAMBIA, G., BENEDETTI PANICI, P., FERRANDINA, G., BATTAGLIA, F., ROSSI, S., BELLANTONE, R., CRUCITTI, F. & MANCUSO, S. (1991). Cathepsin D and epidermal growth factor in human breast cyst fluid. *Br. J. Cancer*, **64**, 965–967.

SCOTT, W.N. & MILLER, W.R. (1990). The mutagenic activity of human breast secretions. *J. Cancer Res. Clin. Oncol.*, **116**, 499–502.

SPYRATOS, F., MAUDELONDE, T., BROUILLET, J.P., BRUNET, M., DEFRENNE, A., ANDRIEU, C., HACENE, K., DESPLACES, A., ROUESSE, J. & ROCHEFORT, H. (1989). Cathepsin D: an independent prognostic factor for metastasis of breast cancer. *Lancet*, **8672**, 1115–1118.

TANDON, A.K., CLARK, G.M., CHAMNESS, G.C., CHIRGWIN, J.M. & MCGUIRE, W.L. (1990). Cathepsin-D and prognosis in breast cancer. *N. Engl. J. Med.*, **322**, 297–302.

THORPE, S.M., ROCHEFORT, H., GARCIA, M., FREISS, G., CHRISTENSEN, I.J., KHALAF, S., PAOLUCCI, F., PAU, B., BRUUN RASMUSSEN, B. & ROSE, C. (1989). Association between high concentrations of $M_r$ 52,000 cathepsin D and poor prognosis in primary human breast cancer. *Cancer Res.*, **49**, 6008–6014.

VIGNON, F., CAPONY, F., CHAMBON, M., FREISS, G., GARCIA, M. & ROCHEFORT, H. (1986). Autocrine growth stimulation of the MCF7 breast cancer cells by the estrogen-regulated 52K protein. *Endocrinology*, **118**, 1537–1545.

WRENSCH, M.R., PETRAKIS, N.L., KING, E.B., MIIKE, R., MASON, L., CHEW, K.L., LEE, M.M., ERNSTER, V.L., HILTON, J.F., SCHWEITZER, R., GOODSON III, W.H. & HUNT, T.K. (1992). Breast cancer incidence in women with abnormal cytology in nipple aspirates of breast fluid. *Am. J. Epidem.*, **135**, 130–141.